# Succinate Dehydrogenase Subunit C Contributes to Mycelial Growth and Development, Stress Response, and Virulence in the Insect Parasitic Fungus Beauveria bassiana

**DOI:** 10.1128/spectrum.02891-22

**Published:** 2022-08-16

**Authors:** Jin-Li Ding, Xiu-Hui Li, Jia-Hui Lei, Ming-Guang Feng, Sheng-Hua Ying

**Affiliations:** a Institute of Microbiology, College of Life Sciences, Zhejiang University, Hangzhou, China; University of Michigan

**Keywords:** succinate dehydrogenase, entomopathogenic fungus, oxidative stress, development, virulence

## Abstract

Succinate dehydrogenase (SDH), also known as respiratory chain complex II, plays a crucial role in energy production in which SdhC functions as an anchored subunit in the inner membrane of mitochondria. In this study, domain annotation analyses revealed that two SdhC domain-containing proteins were present in the filamentous insect-pathogenic fungus Beauveria bassiana, and they were named BbSdhC1 and BbSdhC2, respectively. Only BbSdhC1 localized to mitochondria; hence, this protein is considered the ortholog of SdhC in B. bassiana. Ablation of *BbSdhC1* led to significantly reduced vegetative growth on various nutrients. The Δ*Bbsdhc1* mutant displayed the significantly reduced ATP synthesis and abnormal differentiation under aerial and submerged conditions. Notably, the *BbSdhC1* loss resulted in enhanced intracellular levels of reactive oxygen species (ROS) and impaired growth of mycelia under oxidative stress. Finally, insect bioassays (via cuticle and intrahemocoel injection infection) revealed that disruption of *BbSdhC1* significantly attenuated fungal virulence against the insect hosts. These findings indicate that BbSdhC1 contributes to vegetative growth, resistance to oxidative stress, differentiation, and virulence of B. bassiana due to its roles in energy generation and maintaining the homeostasis of the intracellular ROS levels.

**IMPORTANCE** The electron transport chain (ETC) is critical for energy supply by mediating the electron flow along the mitochondrial membrane. Succinate dehydrogenase (SDH) is also known as complex II in the ETC, in which SdhC is a subunit anchored in mitochondrial membrane. However, the physiological roles of SdhC remain enigmatic in filamentous fungi. In filamentous insect-pathogenic fungus B. bassiana, SdhC is required for maintaining mitochondrial functionality, which is critical for fungal stress response, development, and pathogenicity. These findings improve our understanding of physiological mechanisms of ETC components involved in pathogenicity of the entomopathogenic fungi.

## INTRODUCTION

Beauveria bassiana is a well-investigated filamentous insect-pathogenic fungus for biocontrol of insect pests, and it has become an emerging model fungus for studying the mechanism responsible for the host-pathogen interaction (1, 2). Under natural conditions, infectious fungal cells adhere to the insect cuticle and germinate into invasive germ tubes, which penetrate the host exoskeleton (3). After entering the insect hemocoel, mycelia transform into the form of *in vivo* hyphal bodies (yeastlike cells) through morphological transformation (3, 4). After consuming the nutrients in the hemocoel, the hyphal bodies transform into mycelia, which invade the surrounding tissues and eventually grow out the host exoskeleton (5–7). The growing mycelia produce numerous conidia on the host cadaver for the new infection cycle (8, 9). During the entire infection cycle, B. bassiana utilizes a plethora of nutrients to generate energy for cellular metabolism and development. Mitochondrial architecture and the fission process have been linked to energy generation in B. bassiana (10, 11), but the mechanisms responsible for energy production and the physiological roles of energy metabolism remain largely unknown in the entomopathogenic fungi.

The tricarboxylic acid cycle (TCA cycle), coupled with oxidative phosphorylation (OXPHOS), is the main way to provide energy for cells (12). Succinate dehydrogenase (SDH), also known as respiratory chain complex II, plays a crucial role in the TCA cycle and the electron transport chain (ETC) (13). As an iron-sulfur flavoprotein, SDH catalyzes the energy-dependent oxidation of succinate to fumarate and generates FADH_2_, which transfers electrons to coenzyme Q in the respiratory electron transfer chain (14). This enzyme complex locates in the inner mitochondrial membrane and consists of four subunits, including SdhA, SdhB, SdhC, and SdhD (13, 14). SdhC, as an anchored subunit in the inner membrane of mitochondria, interacts with SdhD (another hydrophobic subunit) and then recruits the SdhA-SdhB complex (hydrophilic unit) to form functional succinate dehydrogenase complex (15). In mammalian cells, mutation of SdhC leads to destabilization of SdhB and SdhD in complex, which significantly compromise the catalytical activities of enzyme complex II (16, 17). In Saccharomyces cerevisiae, mutation of Sdh3 (homolog of SdhC) increases superoxide production and decreases the oxidation resistance of cells (18). In the Botrytis cinerea population (a filamentous plant-pathogenic fungus), mutations of SdhC are associated with fungal resistance to succinate dehydrogenase inhibitors (SDHIs) (19). However, the biological functions of SdhC remain enigmatic in most filamentous fungi.

Here, we identified two proteins with the Sdh_cyt domain (SdhC1 and SdhC2) in B. bassiana, which displayed different subcellular localizations. Only BbSdhC1 was located in mitochondria and was considered the ortholog of yeast SdhC. Its biological roles were genetically characterized via gene disruption and complementation. Disruption of *BbSdhC1* decreased the energy supply and increased the level of reactive oxygen species (ROS) in fungal cells. Additionally, BbSdhC1 contributes to fungal development, stress resistance, and virulence in B. bassiana. More importantly, *BbSdhC1* is under the transcriptional control of the transcription factor BbMbp1. This study first revealed the physiological functions of subunit SdhC in the succinate dehydrogenase complex for filamentous fungi.

## RESULTS

### Characterization, subcellular location, and molecular manipulation of *BbSdhC*.

**FIG 1:**
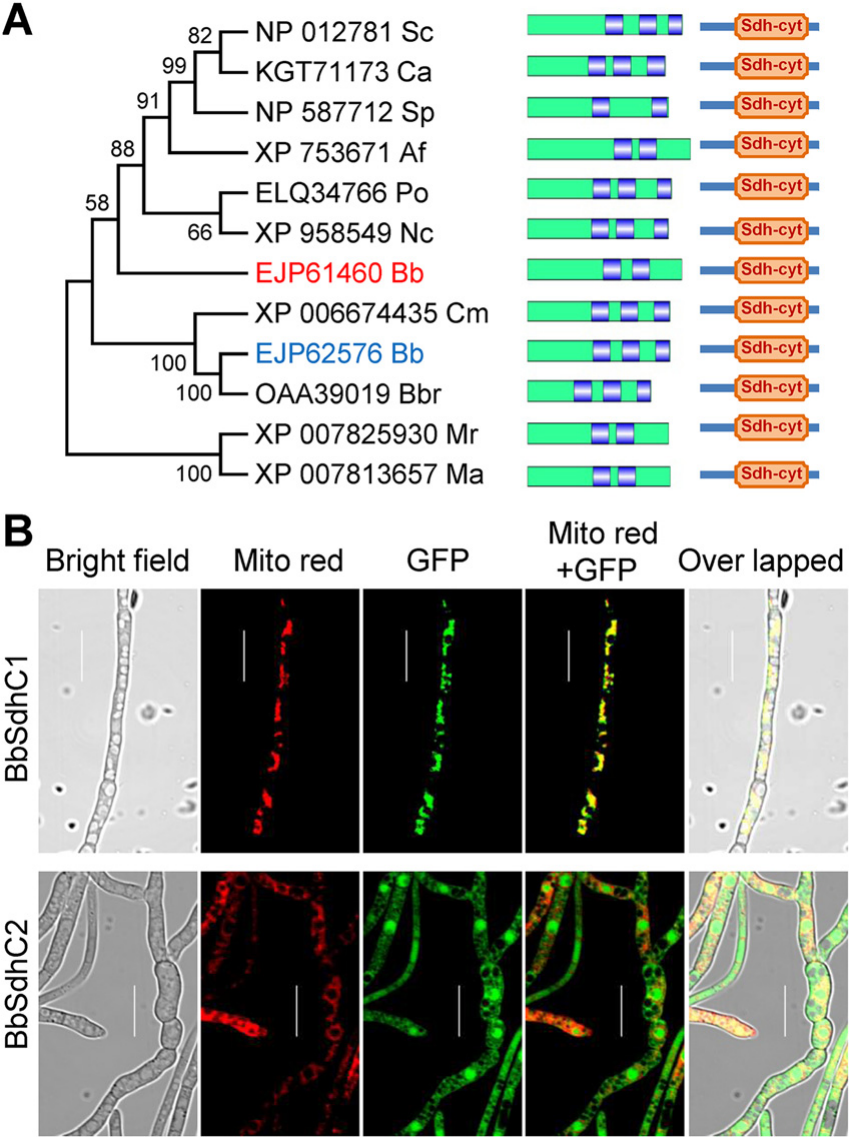
Bioinformatic analyses and subcellular localization of SdhC in B. bassiana. (A) Relationships among the SdhC homologs were constructed by the neighbor-joining method, and the bootstrap values from 1,000 replicates are shown at nodes. Only B. bassiana has two homologous proteins. Abbreviations for fungal species followed by GenBank accession numbers of corresponding genes are shown as follows: Sc, Saccharomyces cerevisiae S288C; Ca, Candida albicans 12C; Sp, Schizosaccharomyces pombe; Af, Aspergillus fumigatus Af293; Po, Pyricularia oryzae Y34; Nc, Neurospora crassa OR74A; Bb, Beauveria bassiana ARSEF 2860; Cm, Cordyceps militaris CM01; Bbr, Beauveria brongniartii RCEF 3172; Mr, Metarhizium robertsii ARSEF 23; and Ma, Metarhizium acridum CQMa 102. All SdhC proteins contain an Sdh-cyt domain at the C termini. Transmembrane helixes were indicated by the blue area in green bar (representing the SdhC sequence). (B) Subcellular localization of SdhC homologs in B. bassiana. The 2-day-old mycelia were harvested from SDB, and LSCM images for subcellular localization of the GFP-SdhC fusion protein were taken for mycelia stained with mitochondrion-specific dye (shown in red). Scale bars, 10 μm.

Based on the BLAST research with yeast Sdh3 (GenPept accession no. NP_012781) as a query, two highly related homologs (GenPept accession nos. EJP61460 [E value, 5e-04] and EJP62576 [E value, 1e-08]) were identified in B. bassiana, and they were named BbSdhC1 and BbSdhC2, respectively. Domain annotation analyses indicated that both BbSdhC1 and BbSdhC2 contained a domain of Sdh_cyt (SdhC) (PF01127.22). As shown in Fig. 1A, BbSdhC was much more closely related to those of the filamentous fungi than to those of yeasts and showed more similarity to those of entomopathogenic fungi. BbSdhC1 and BbSdhC2 had two and three transmembrane helixes, respectively. No correlation was observed between the number of transmembrane helixes and fungal lifestyle, i.e., saprobes, plant, animal, or insect pathogens. B. bassiana had two BbSdhC proteins, which significantly differed from most fungal species. Multiple-sequence alignment analysis indicated that there were conserved regions in the SdhC-containing proteins from fungal species (see Fig. S1 in the supplemental material).

To visualize the subcellular localization of BbSdhC proteins, the indicated gene was fused to green fluorescence protein gene as a reporter. As shown in Fig. 1B, green globular and reticulated signals were observed for the GFP-tagged BbSdhC1, whereas only globular signals were observed for the GFP-tagged BbSdhC2. After staining with MitoTracker Red CMXRos (emitting red signals), the green signals for BbSdhC1 were colocalized with the red signals. The green signals for BbSdhC2 had no significant overlap with the red signals (Fig. 1B). These results indicated that BbSdhC1 localizes in mitochondria and BbSdhC2 did not. Thus, BbSdhC1 was considered the succinate dehydrogenase subunit C in B. bassiana and was genetically analyzed in a subsequent study.

The open reading frame (ORF) sequence of *BbSdhC1* was 820 bp long, with 2 introns in the genomic sequence, and it coded for a protein with 196 amino acids. The disruption and complementation strains of *BbSdhC1* were constructed by homologous recombination and ectopic integration, respectively. PCR and Southern blotting were used to determine the correct recombination events in the gene disruption and complementation mutant strains (Fig. S2A through C). These results indicated that the homolog of yeast SdhC was determined in B. bassiana, and the gene disruption and complementation mutant strains were successfully obtained.

### BbSdhC1 contributes to vegetative growth.

**FIG 2:**
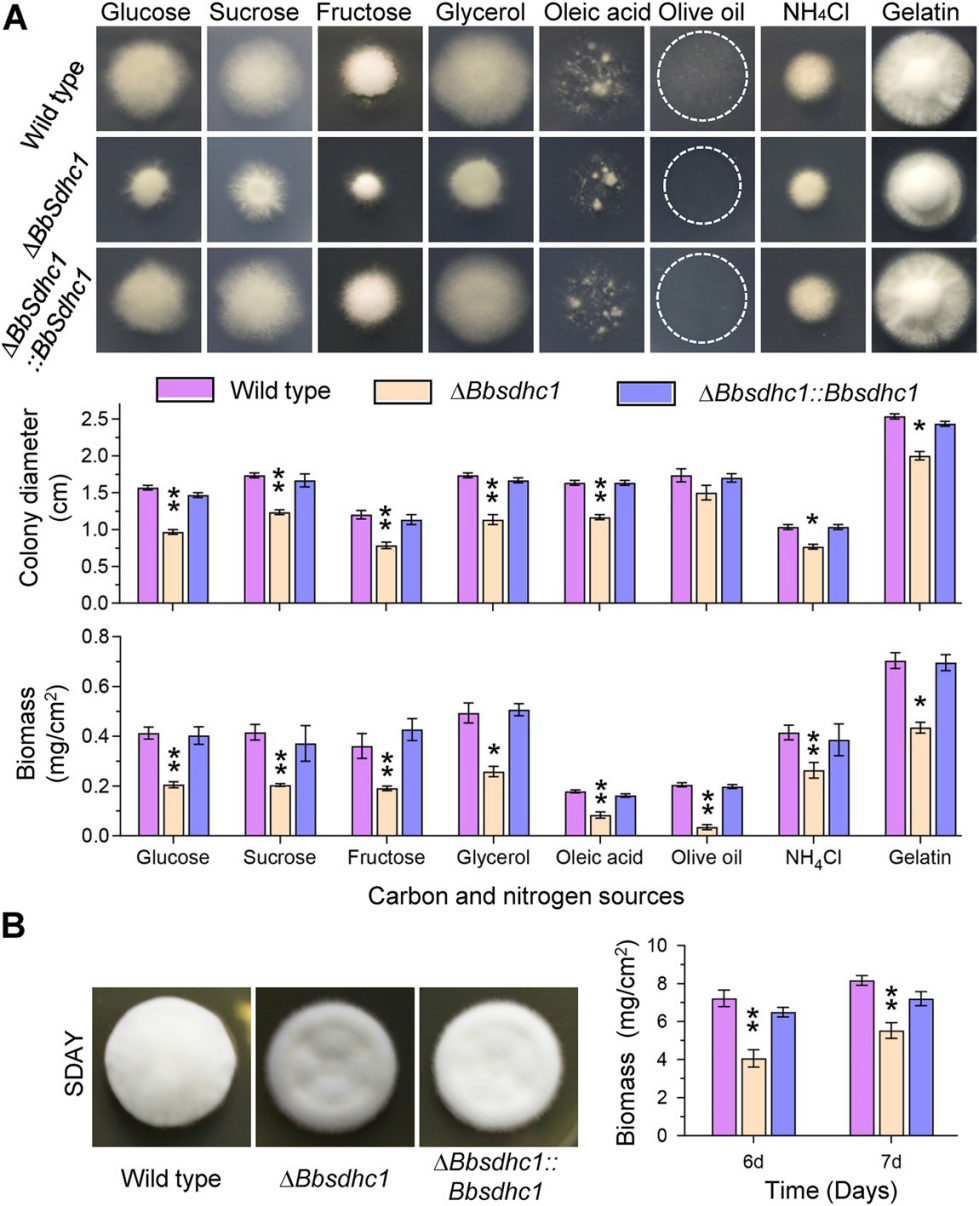
Impacts of the *BbSdhC1* loss on vegetative growth of B. bassiana. (A) Images and diameter of fungal colonies. Aliquots of 1-μL suspensions (10^6^ conidia/mL) were inoculated on the plates of modified CZA, which contained different carbon and nitrogen sources (glucose, sucrose, fructose, glycerol, oleic acid, olive oil, NH_4_Cl, and gelatin). After an incubation of 7 days at 25°C, colony diameter was measured. Cycles indicate the margin of the colony formed on olive oil. Mycelial biomass was quantified by incubating 100 μL conidial suspension on SDAY plates for 7 days. (B) Colony morphology and biomass on SDAY plate. The morphology for 7-day-old colonies was determined by inoculating conidial suspension (1 μL, 10^6^ conidia/mL) on SDAY plates. Mycelial biomass was quantified by incubating 100 μL conidial suspension on SDAY plates for 6 and 7 days. Asterisks on the columns indicate a significant difference between the Δ*Bbsdhc1* mutant and the wild-type or complemented strains (Student’s *t* test; *, *P* < 0.05; **, *P* < 0.01). Error bars, standard deviation.

To determine the roles of BbSdhC1 in nutrient utilization, fungal growth was evaluated on different carbon or nitrogen sources (Fig. 2A). After a 7-day incubation at 25°C, the disruption mutant showed a significant reduction in colony diameter and biomass. On the Czapek-Dox (CZA) plate, the colony diameter of the Δ*Bbsdhc1* mutant decreased 38.27% compared with that of the wild-type strain, and on all modified CZAs, it decreased 13.46 to 34.72%; the decrease in mycelial biomass ranged from 36.5% to 82.9%. On a rich Sabouraud dextrose agar (SDAY) plate, the colony diameter of the gene disruption mutant was almost the same as that of the wild type. However, the Δ*Bbsdhc1* mutant developed into a thin colony compared with the wild-type strain. In addition, the colony biomass of Δ*Bbsdhc1* mutant was significantly less than that for the wild-type and complementation mutant strains (Fig. 2B). These data indicated that BbSdhC1 contributes to fungal vegetative growth.

### BbSdhC1 is required for asexual development.

**FIG 3:**
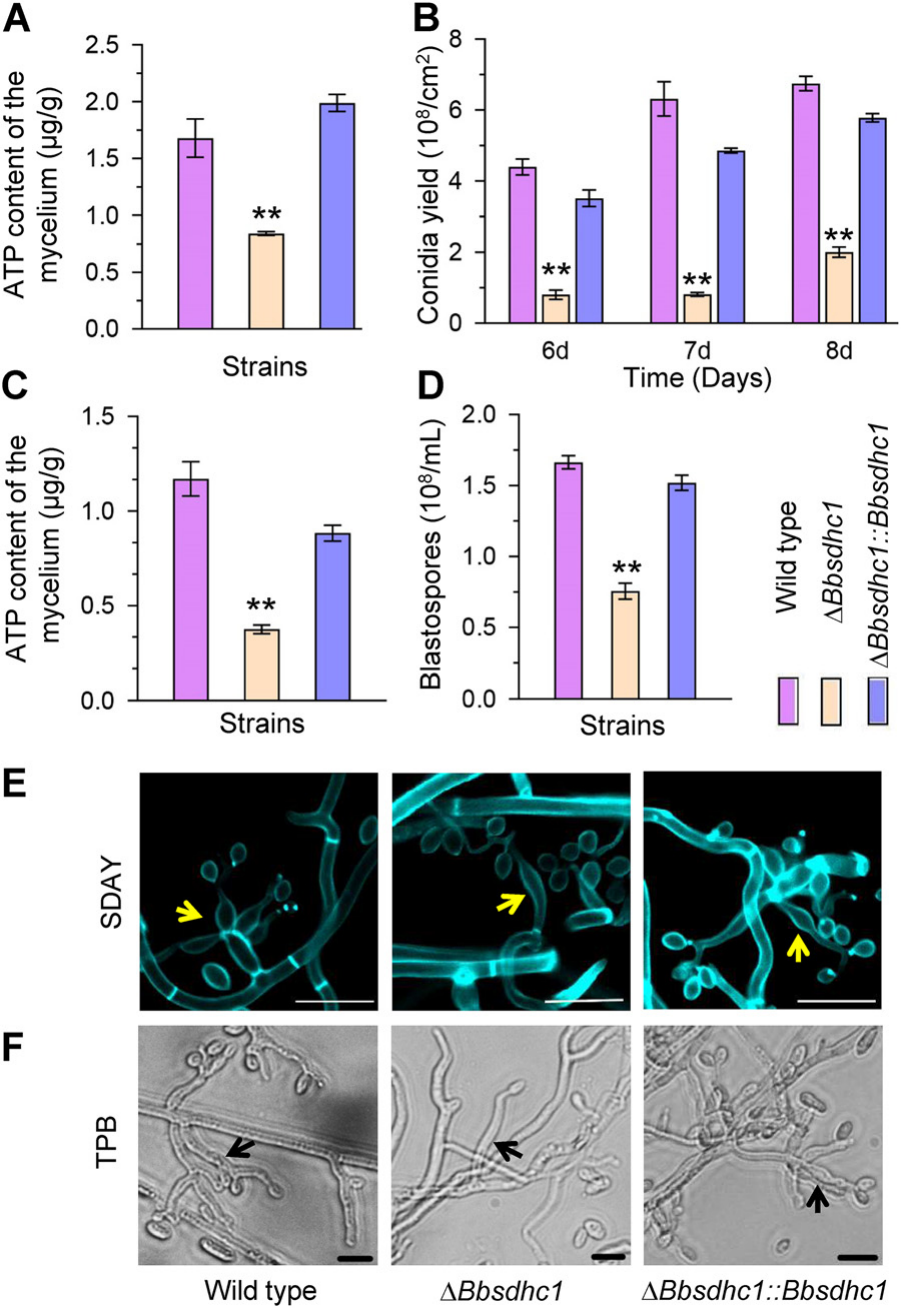
BbSdhC1 is required for asexual development in B. bassiana. Conidial suspensions were inoculated on SDAY plates and cultured at 25°C. (A) ATP content quantified in mycelia cultured for 3.5 days. (B) Conidial production was examined at 6, 7, and 8 days postincubation. (C and D) Under submerged conditions, conidial suspension was inoculated into SDB and cultured at 25°C for 3 days, and then mycelial ATP content mycelia (C) and blastospore production (D) were quantified. (E) On SDAY plates, the wild-type and complementation strains generated normal conidiophores (indicated with yellow arrows), and the Δ*Bbsdhc1* strain did not. Fungal cells were stained with calcofluor white and examined under a fluorescence microscope. Scale bars, 5 μm. (F) In TPB medium, disruption of *Bbsdhc1* caused a slight change in morphology of the blastospore-producing structures (indicated with black arrows) compared with the wild-type and complementation strains. Scale bars, 10 μm. Asterisks on columns indicate the significant difference between the wild-type or complemented strains (Student’s *t* test; *, *P* < 0.05; **, *P* < 0.01). Error bars, standard deviation.

At 4 days postincubation under aerial conditions (Fig. 3A), the ATP content in the Δ*Bbsdhc1* mutant was 0.83 ± 0.02 μg/g (mean ± standard deviation [SD]), with approximate 50.0% reduction compared with that of the wild-type strain. The Δ*Bbsdhc1* mutant exhibited a dramatic decrease in conidial yield on SDAY plates. At 6, 7, and 8 days postincubation, the reductions in conidial yield were 81.7, 87.1, and 70.3%, respectively (Fig. 3B). Microscopic examination indicated that the wild-type and complemented strains produced plentiful conidia-generating structures at 4 days postincubation. However, the Δ*Bbsdhc1* mutant strain produced fewer conidia-forming structures, which displayed a distorted appearance (Fig. 3E).

Fungal development was evaluated in trehalose-peptone broth (TPB) mimicking insect hemolymphs. The *BbSdhC1* loss resulted in a significant reduction in ATP content (Fig. 3C). The ATP content in the Δ*Bbsdhc1* mutant was 0.38 ± 0.04 μg/g, with an approximate 67.9% reduction compared with that of the wild-type strain (1.17 ± 0.16 μg/g). As shown in Fig. 3D, blastospore concentrations for Δ*Bbsdhc1* were 0.76 × 10^8^ ± 0.08 × 10^8^ spores mL^−1^ (mean ± SD), with a decrease of 54.6% in comparison to that of the wild-type strain (1.67 × 10^8^ ± 0.07 × 10^8^ spores mL^−1^). The wild-type and complemented strains generated numerous branchlike spore formation structures on the mycelia in TPB, and they were abnormal in morphology and number in the Δ*Bbsdhc1* mutant (Fig. 3F). These results indicate that BbSdhC1 plays an important role in formation of conidia and blastospores on aerial surfaces and in liquid environments, respectively.

### BbSdhC1 plays an important role in fungal responses to oxidative stress.

**FIG 4:**
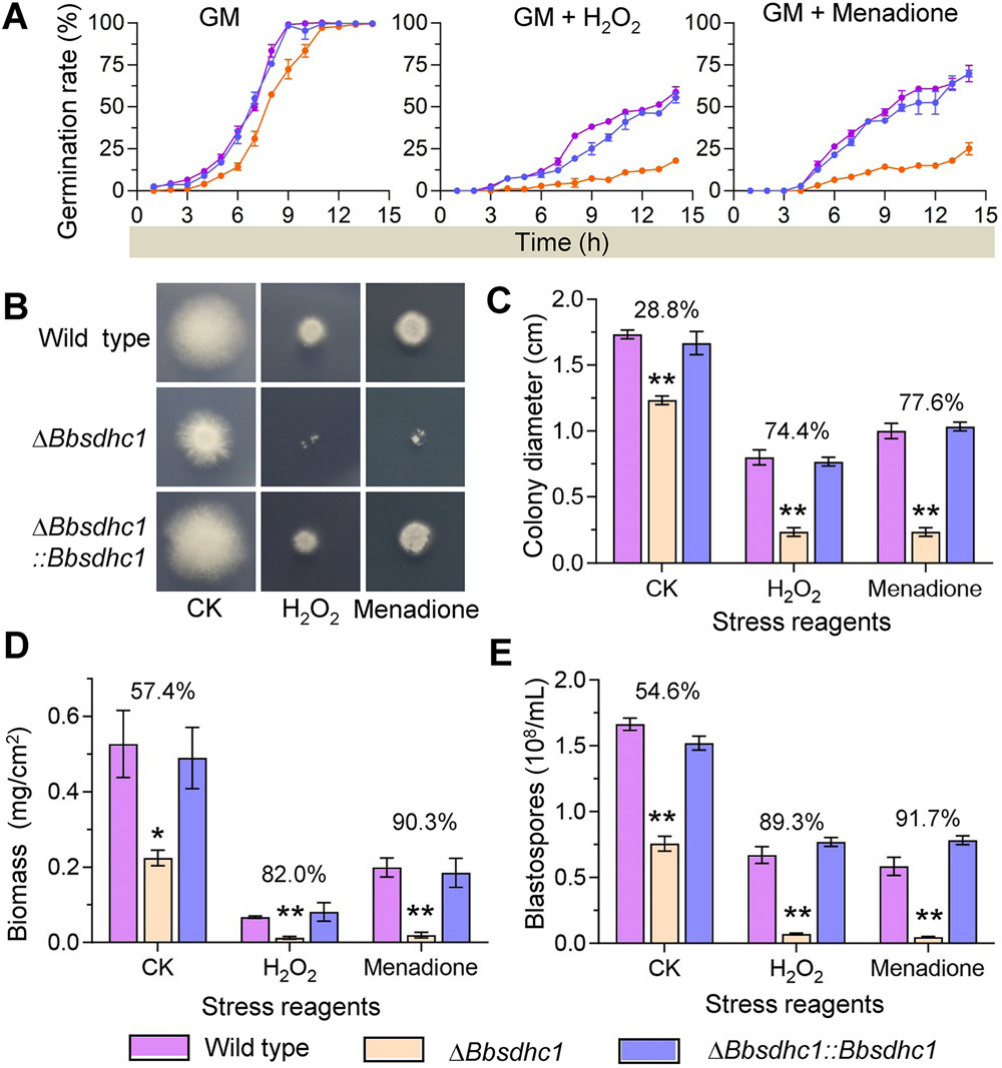
BbSdhC1 is required for B. bassiana responses to oxidative stress. (A) Conidial germination under oxidative stress. Conidial suspension of the indicated strains were inoculated on germination medium (GM) supplemented with H_2_O_2_ and menadione and then incubated at 25°C. Germination percentage (%) was determined every 2 h for 14 h. GM without stress chemical was used as control. (B and C) The effect of oxidative stress on vegetative growth was evaluated at 7 days postincubation. The colony diameters were measured on the Czapek-Dox plates containing either 2 mM H_2_O_2_ or 0.02 mM menadione, using Czapek-Dox plates as controls. (D) Biomass of the colony. (E) Effect of oxidative stress blastospore production under submerged condition. Fungal strain was cultured in TBP medium included with 6 mM H_2_O_2_ or 0.06 mM menadione, using TBP medium as control. Three days later, the blastospore yield was measured. Asterisks on the columns indicate a significant difference between the Δ*Bbsdhc1* mutant and the wild-type or complemented strain (Student’s *t* test; *, *P* < 0.05; **, *P* < 0.01). The percentage on the column group indicates the reduction of the phenotypic measurement in Δ*Bbsdhc1* mutant strain compared with the wild-type strain. Error bars, standard deviation.

As shown in Fig. 4A, under nonstressful conditions, the median germination time of Δ*Bbsdhc1* was 8.14 ± 0.35 h, which was 2 h later than that of wild-type strain (6.08 ± 0.09 h), and there was no significant difference in germination rate between the disruptant and wild-type strains at 14 h postincubation. On plates supplemented with H_2_O_2_, the germination rates for the wild-type and Δ*Bbsdhc1* mutant strains were 58.7% ± 3.1% and 18.0% ± 1.0% at 14 h postincubation, respectively. Under menadione stress, the germination percentages for the wild-type and Δ*Bbsdhc1* mutant strains were 70.0 ± 4.9% and 25.0 ± 3.5%, respectively.

Vegetative growth under oxidative stress was determined by evaluating radial growth rates and biomass on CZA media supplemented with H_2_O_2_ and menadione (Fig. 4B). After a 7-day incubation at 25°C, the Δ*Bbsdhc1* mutant showed severe growth defects. On H_2_O_2_ and menadione plates, the colony diameter of Δ*Bbsdhc1* decreased by approximately 75% and 80%, respectively, compared with that of the wild-type strain. However, on the control plates, the colony diameter of the Δ*Bbsdhc1* mutant strain only decreased 28.8% in comparison to the wild type (Fig. 4C). The biomass of the Δ*Bbsdhc1* mutant strain decreased 57.4% on CZA plates compared with that of the wild-type strain. The reductions in biomass of Δ*BbsdhC1* on the H_2_O_2_ and menadione plates were approximately 80% and 90%, respectively (Fig. 4D).

As mentioned above, disruption of *BbSdhC1* caused an approximate 50% reduction in blastospore production (Fig. 3D). Oxidative stress exacerbated this defect in the Δ*Bbsdhc1* mutant strain (Fig. 4E). After including H_2_O_2_ and menadione into TBP medium, the blastospore concentration of the Δ*Bbsdhc1* strain decreased 89.30% and 91.73%, respectively, compared with that of the wild-type strain. There results suggested that BbSdhC1 is required for conidial germination, vegetative growth, and blastospore formation under oxidative stress.

### BbSdhC1 maintains the homeostasis of intracellular ROS.

**FIG 5:**
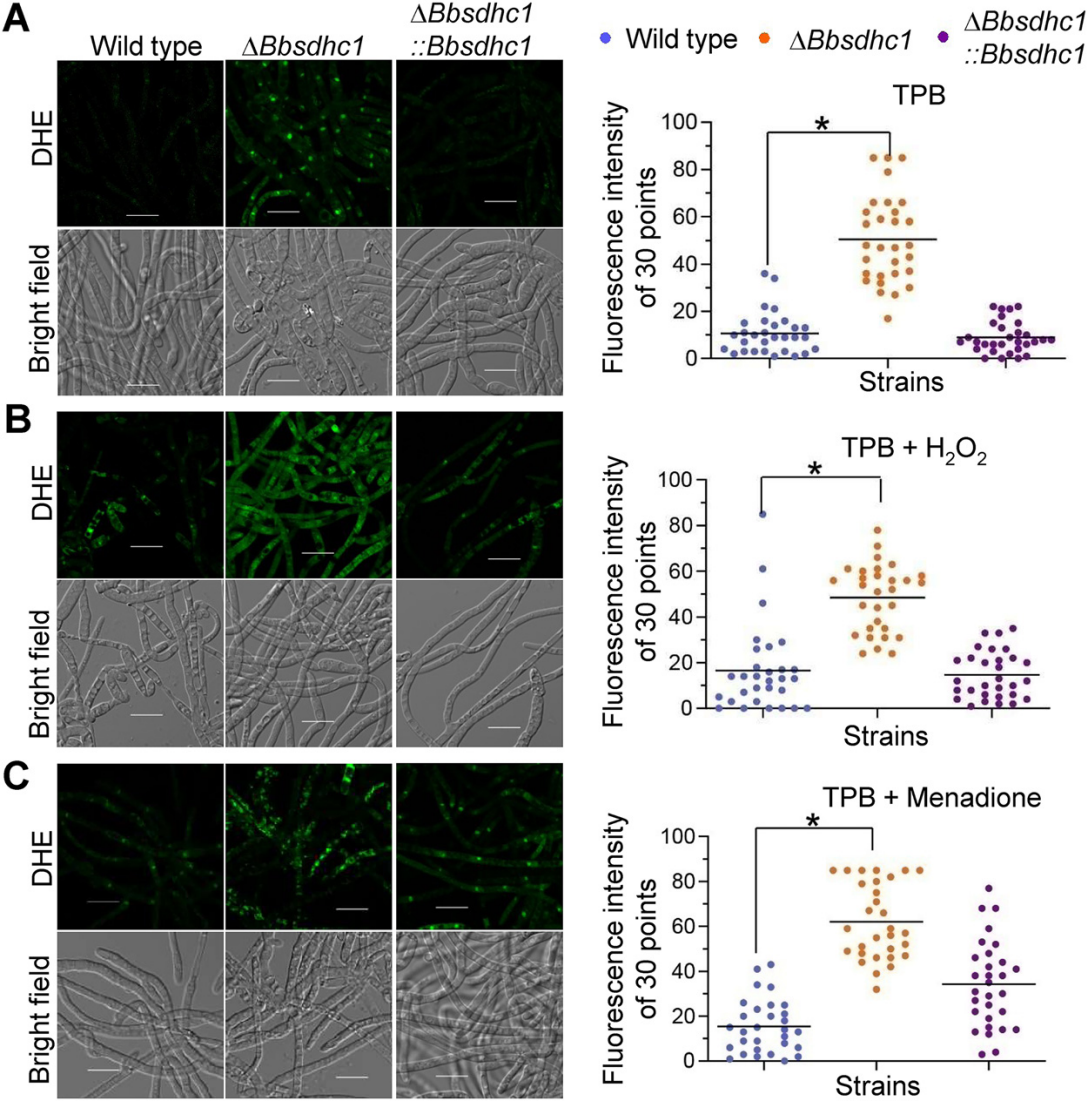
ROS content in the submerged mycelia. (A to C) On the basis of TPB medium (A), stress reagents H_2_O_2_ (B) and menadione (C) were included into medium to establish oxidative stress. Conidia of the indicated strains were inoculated into culture broth and cultured at 25°C for 2 days. ROS in mycelia were visualized by staining with fluorochrome DHE. Fluorescence intensity was recorded for 30 sampled mycelia. Its difference between two strains was determined by Mann-Whitney test and indicated by an asterisk when the *P* value was less than 0.05. Error bars, standard deviation.

As shown in Fig. 5, the loss of BbSdhC1 increased the intracellular content of ROS, which could be enhanced by oxidative stress. In TPB medium, DHE (superoxide-specific molecular probes) fluorescence intensity of 30 points in Δ*Bbsdhc1* mycelium (50.5 ± 18.5) increased by approximate 4 times compared with that the wild-type strain (10.6 ± 8.74) (Fig. 5A). In broth supplemented with H_2_O_2_, fluorescence intensity in the wild-type strain increased 55.8% compared with that on control plates. The fluorescence intensity of the Δ*Bbsdhc1* mutant strains was significantly higher than that in the wild-type strain (Fig. 5B). Under menadione stress, the fluorescence intensity for the wild-type and Δ*Bbsdhc1* mutant strains was 15.5 ± 11.9 and 62.1 ± 16.8, respectively, in which significant difference was observed (Fig. 5C). Additionally, the Δ*Bbsdhc1* mutant exhibited a significant increase in ROS content when growing on SDAY plates (Fig. S3). At 4 days postincubation, the fluorescence intensity in Δ*Bbsdhc1* mycelium was increased 61.43% compared with that in the wild-type strain. These data indicated that BbSdhC1 significantly contributes to maintaining the homeostasis of ROS content during differentiation and under oxidative stress.

### Essential roles of BbSdhC1 in fungal virulence.

**FIG 6:**
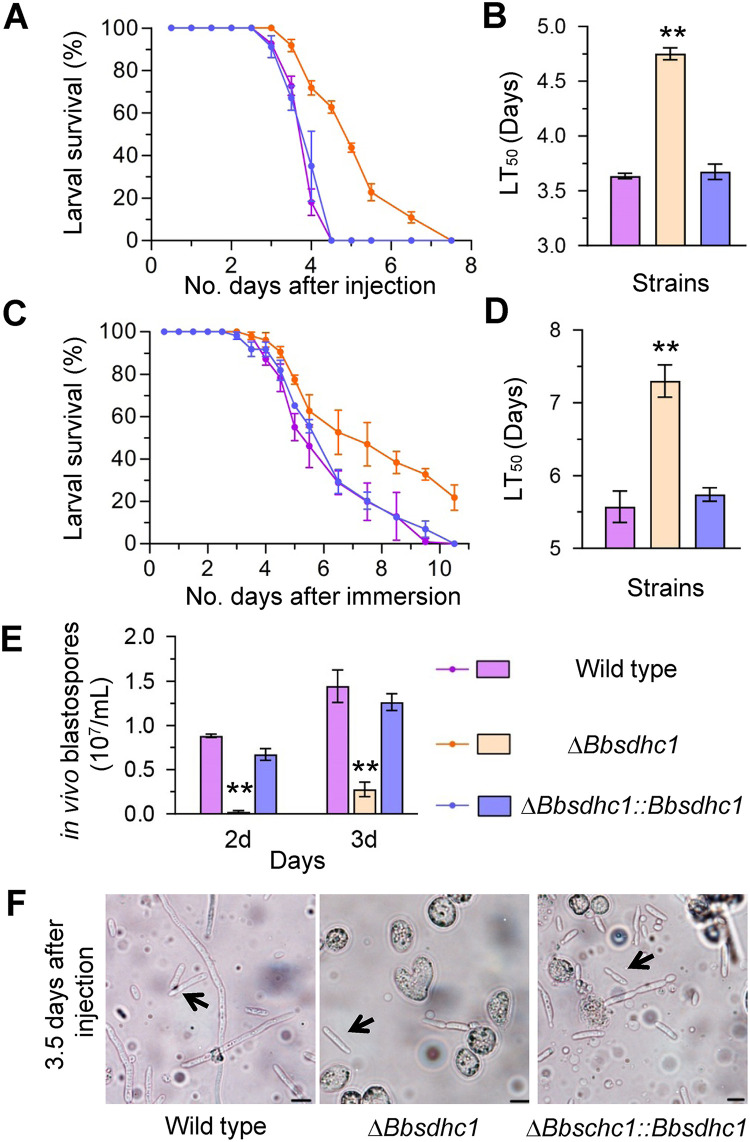
BbSdhC1 contributes to B. bassiana virulence. In a bioassay, survival trends were recorded for G. mellonella larvae after topical application (A) of a 10^7^ conidia/mL suspension for cuticle infection and intrahemocoel injection (C) of ~500 conidia, respectively. Median lethal time (LT_50_) for topical application (B) and intrahemocoel injection (D) were calculated by probit analyses. Additionally, the BbSdhC1 loss significantly compromised fungal dimorphism into hyphal bodies in host hemocoel (E) but did not affect the morphologies of the hyphal bodies (F). The arrows indicate the *in vivo* hyphal body (scale bars, 10 μm). Asterisks on the columns indicate a significant difference between the gene disruption mutant and the wild-type or complemented strain (Student's *t* test; *, *P* < 0.05; **, *P* < 0.01). Error bars, standard deviation.

Two types of bioassay methods were used to evaluate fungal virulence against Galleria mellonella larvae (18). In cuticle inoculation bioassay, the median lethal time (LT_50_) value of Δ*BbsdhC1* was 7.30 ± 0.31 days (mean ± SD), delayed for 1.73 ± 0.46 days compared with that of the wild type (5.57 ± 0.31 days), with a delay of 31% (Fig. 6A and B). In the intrahemocoel injection bioassay, the LT_50_ for the Δ*Bbsdhc1* mutant was 4.75 ± 0.08 days, significantly different from that of the wild-type strain (3.63 ± 0.03 days), with a delay of 30% (Fig. 6C and D). Notably, disruption of *BbSdhC1* led to a significant reduction in the yield of *in vivo* hyphal bodies (Fig. 6E). The Δ*Bbsdhc1* mutant only produced 0.02 × 10^7^ ± 0.02 × 10^7^ spores mL^−1^ in *in vivo* hyphal bodies at 2 days postinfection, with approximately 97.36% reduction compared with that of the wild-type strain (0.88 × 10^7^ ± 0.02 × 10^7^ spores mL^−1^). The spore yield of the Δ*Bbsdhc1* strain increased at 3 days postinfection but still displayed an approximate 80% reduction compared with that of the wild-type strain. However, there was no significant difference in the morphology of the *in vivo* blastospore between the gene disruption and wild-type or complemented strains (Fig. 6F). These data indicated that the *BbSdhC1* loss resulted in impaired pathogenic growth, which ultimately weakened fungal virulence.

### BbMbp1 contributes to fungal oxidation resistance and interacts with the *BbSdhC1* promoter.

**FIG 7:**
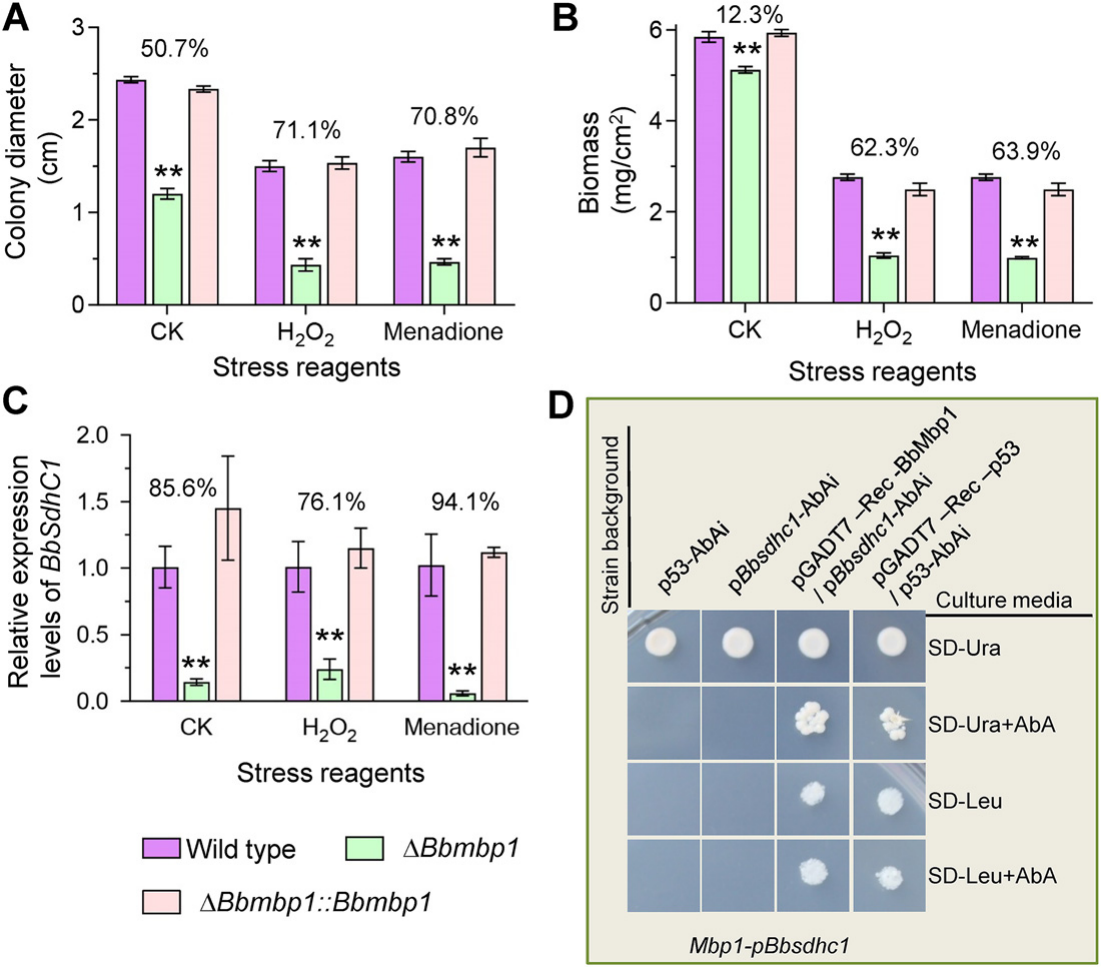
Oxidative stress responsive transcription factor BbMbp1 interacts with the *BbSdhC1* promoter. (A) Colony diameters were measured for the wild-type, Δ*Bbmbp1*, and complemented strains on CZA plates containing either 2 mM H_2_O_2_ or 0.02 mM menadione, using CZA plate as control. (B) Biomass under submerged condition. The indicated strain was cultured in TBP medium with 6 mM H_2_O_2_ or 0.06 mM menadione, using TBP medium as control (CK). After 3 days, the biomass was measured. (C) qRT-PCR was used to analyze the relative expression level of *BbSdhC1* under oxidative stresses caused by H_2_O_2_ and menadione. CZA without stress agent was used as control. Asterisks on the columns indicate a significant difference between the Δ*Bbsdhc1* mutant and the wild-type or complemented strain (Student’s *t* test; *, *P* < 0.05; **, *P* < 0.01). The percentage on the column group indicates the reduction of the phenotypic measurement in Δ*Bbsdhc1* mutant strain compared with the wild-type strain. Error bars, standard deviation of three replicates. (D) Yeast one-hybrid assay for the interaction of BbMbp1 with the promoter fragments of *BbSdhC1*. The promoter region of *Bbsdhc1* and BbMbp1-coding sequence were cloned in bait vector (pAbAi) and expression vector (pGADT7-Rec AD), respectively. The interaction was confirmed on SD−Ura or synthetic dextrose medium + aureobasidin A (SD + Aba) medium. A negative strain (p53-AbAi) and a positive strain (pGADT7-Rec-p53/p53-AbAi) were used as controls.

In B. bassiana, a DNA-binding protein Mbp1 (BbMbp1) contributes to fungal development. Comparative transcriptomic analyses indicated that BbMbp1 mediates transcription of *BbSdhC1* (9). We suggested that BbMbp1 might play a role in the response of B. bassiana to oxidative stress. As shown in Fig. 7A and B, the loss of BbMbp1 significantly impaired fungal resistance to oxidative stress. In the H_2_O_2_- and menadione-added media, the colony diameter of Δ*Bbmbp1* decreased by approximately 70% compared with that of the wild-type strain. On the control plates (CZA), the colony diameter of the Δ*Bbmbp1* mutant strain only decreased by roughly 50%. In TPB supplemented with H_2_O_2_ and menadione, biomass of the Δ*Bbmbp1* mutant was significantly decreased by approximately 63% compared with that of the wild-type strain. In the control broth (TPB), the biomass of the Δ*Bbmbp1* mutant strain only decreased by 12.3%.

As shown in Fig. 7C, quantitative real-time PCR (qRT-PCR) analyses indicated that the expression level of BbSdhC1 was significantly repressed in the Δ*Bbmbp1* mutant strain. The reduction on the control plate was approximately 85%. Under H_2_O_2_ and menadione stresses, the reductions were approximately 75% and 95%, respectively. The yeast one-hybrid (Y1H) test was used to determine the requirement of BbMbp1 for transcription of *BbSdhC1* (Fig. 7D). The transformants with the bait vector grew well on the synthetic dextrose medium lacking uridine (SD–ura) medium, which indicated successful construction of the reporter strains. After introducing the expressing vector into the reporter strain, the yeast cells grew well on the selective medium, which indicated BbMbp1 activates the transcription of *BbSdhC1*. No canonical MluI-binding sites (5′-ACGCGT-3′) were detected in the promoter region of *BbSdhC1*; however, similar sequences (e.g., 5′-ACGCGA-3′) could be found (Fig. S4). These results indicated that *BbSdhC1* functions as a target gene of transcription factor BbMbp1 during fungal development and response to oxidative stress.

## DISCUSSION

Succinate dehydrogenase (SDH) is an enzyme complex in the electron transport chain in which subunit C (SdhC) functions as an anchored protein in the inner membrane of mitochondria (17). Unlike most fungi, B. bassiana contains two SdhC subunits in which only BbSdhC1 localizes in mitochondria. In the present study, the biological roles of BbSdhC1 were genetically characterized in filamentous fungi, and our results indicated that BbSdhC1 contributes to mitochondrial functionality, which is required for fungal development, oxidation tolerance, and virulence.

Virulence is critical for efficacy of the entomopathogenic fungi as biocontrol agents (20, 21) and results from the combined effects of the host-pathogen interactions (22). During the infection cycle of B. bassiana, fungal cells suffer from oxidative stress. In the host hemocoel, B. bassiana encounters reactive oxygen species (ROS) (e.g., O_2_^−^) produced by the host immune defense (23). BbSdhC1 contributes to fungal resistance to oxidative stress. Cells lacking BbSdhC1 accumulate much more ROS and display enhanced sensitivity to oxidative stress. Similar results were observed in other organisms. The SDH complex mediates electron transfer to the ubiquinol cytochrome *c* oxidoreductase (complex III). In budding yeast, mutation of Sdh3 (homolog of SdhC) resulted in an increased level of ROS and decreased resistance to oxidative stress (18). In human cells, the SDH complex is already known to be a source of ROS production (14), and its mutation decreases cellular resistance to oxidative stress and leads to many diseases induced by high levels of ROS (24, 25). These findings suggest that subunit SdhC is required for maintaining the electron flow, which prevents additional superoxide production. In Magnaporthe oryzae, a mitochondrial protein electron-transferring flavoprotein (ETF) mediates delivery of electrons to ubiquinone in the electron transport chain (ETC) and contributes to fungal resistance to oxidative stress (26). This reinforces that the homeostasis of the electron flow in ETC is essential for fungal response to oxidative stress.

B. bassiana undergoes dimorphic transition from hyphal form into yeastlike form, which is critical for its virulence (12, 16). BbSdhC1 is required for ATP formation during blastospore development under submerged conditions. In B. bassiana, mitofilin (a protein of the mitochondrial inner membrane) and the fission-related proteins (Fis1, Mdv1, and Dnm1) contribute to ATP generation. Their losses significantly impair fungal development into blastospores (10, 11). It is reasoned that SdhC1 is critical for energy production by mitochondria, which is involved in blastospore formation. In addition, oxidative stress exacerbates the defect in blastospore development of the Δ*BbsdhC1* mutant. As discussed above, BbSdhC1 is required for maintaining the homeostasis of intracellular ROS levels, which is critical for fungal development and other physiologies (27). This suggests that the exacerbated defects might be from secondary effects caused by the increased ROS level. The impaired development under oxidative stress is attributed to the very low production of *in vivo* hyphal bodies in the Δ*Bbsdhc1* mutant, which ultimately weakens fungal virulence. In addition, BbSdhC1 contributes to vegetative growth on various nutrients. Therefore, BbSdhC1 contributes to B. bassiana virulence by its roles in fungal growth and development under oxidative stress. A series of physiological processes have been linked to the dimorphic change in B. bassiana under oxidative stress, such as autophagy (28), cell cycle (9), and signal transduction (29). This study first highlights a component of the ETC involved in fungal development under environmental stress. After killing the host, B. bassiana produces plentiful conidia for its survival and dispersal in the ecosystem (30). BbSdhC1 is also required for conidiation due to its role in energy generation as a component in the ETC. In addition, the BbSdhC1-mediated energy supply is required for morphologies of spore-producing structures under aerial and submerged conditions.

The MluI cell cycle box-binding complex is essential for cell development in yeast. In the protein complex, the Mbp1 protein acts as a DNA-binding protein and plays pleiotropic roles in physiological processes (31). In B. bassiana, BbMbp1, together with transcription activator Swi6, mediates comprehensive transcriptome in fungal development, including the transcriptional activation of BbSdhC1 (9). Additionally, BbMbp1 contributes to fungal response to oxidative stress. These results suggest that in terms of physiological functions, BbMbp1 and BbSdhC1 are in the same pathway. *BbSdhC1* was identified as a downstream target of the transcription factor BbMbp1 in the Y1H system. Thus, this suggests that BbMbp1 plays an important role in fungal development and oxidation tolerance, which is partially due to the contributions of the Mbp1-SdhC pathway (MSP). Ubiquinone (CoQ) is an important coenzyme molecule of the ETC, and its synthesis is differentially regulated by Mig1, Rtg3, and HapII in yeast (32). Thus, this study highlights a transcription factor that significantly contributes to transcriptional regulation of the architectural protein in ETC.

In summary, we have identified two SdhC domain-containing proteins in B. bassiana with different subcellular localizations in which BbSdhC1 localizes in mitochondria. Our data revealed that BbSdhC1 is required for energy generation and maintaining the homeostasis of intracellular ROS level, which is essential for vegetative growth, stress response, differentiation, and virulence. The virulence contribution of BbSdhC1 is due to its participation in fungal development and stress resistance during the entire infection cycle. Notably, BbSdhC1, as an ETC gene, is under the transcriptional control of Mbp1, a component of the MluI cell cycle box-binding complex. This study initiates our understanding of the involvements of electron transfer chain into the lifestyle of the insect-pathogenic fungi.

## MATERIALS AND METHODS

### Strains and culturing conditions.

Microbial strains were cultured as described previously (28). The wild type of B. bassiana ARSEF2860 (WT) was routinely maintained on Sabouraud dextrose agar (SDAY; 4% glucose, 1% peptone, 1% yeast, and 1.5% agar). Escherichia coli DH5α (Invitrogen, Carlsbad, CA, USA) was cultured in Luria-Bertani medium for plasmid propagation, using an appropriate antibiotic as the selection reagent. In fungal transformation, Agrobacterium tumefaciens AGL-1 was grown in yeast extract broth (0.5% sucrose, 1% peptone, 0.1% yeast extract, and 0.05% MgSO_4_ [wt/vol]). Czapek-Dox agar (CZA; 3% sucrose, 0.3% NaNO_3_, 0.05% KCl, 0.1% K_2_HPO_4_, 0.05% MgSO_4_, 0.001% FeSO_4_, and 1.5% agar) was used as a defined medium in this study.

### Bioinformatic analyses of SdhC *in* B. bassiana.

We used the sequence of yeast Sdh3 protein (GenPept accession no. NP_012781) as a query sequence to identify the homolog in the B. bassiana genome (33) by a local BLAST program. Two resulting homologs (GenPept accession nos. EJP61460 and EJP62576) were named BbSdhC1 and BbSdhC2, respectively. The homologs in other fungi were downloaded from the NCBI database, and their phylogenetic relationship was analyzed with MEGA software using the neighbor-joining method. The domain features of these homologs were recognized through an online portal, SMART (http://smart.embl-heidelberg.de/).

### Subcellular localization of SdhC1and SdhC2 in B. bassiana.

Plasmid construction was performed as described previously (34). Briefly, the coding sequence of each gene was amplified with the respective primers (PL_x_F/PL_x_R) (X, BbSdhC1 and BbSdhC2) using cDNA as the template. The resultant gene fragments were fused to the 5′ terminus of green fluorescence protein gene in the vector p0380-gfp-myc-bar (35).The resultant p0380-gfp-X-bar was integrated into the WT strain via *Agrobacterium*-mediated transformation. Putative transformants were screened on the selection plates with phosphinothricin (200 μg/mL) and confirmed under a fluorescence microscope. The conidia of transformants were inoculated in SDB (SDAY without agar) and incubated at 25°C for 2 days. Hyphal samples collected from each culture were stained with MitoTracker Red (Invitrogen, Shanghai, China). Fluorescent signals were visualized under a laser scanning confocal microscope (LSCM).

### Target gene replacement via homologous recombination.

Gene disruption and complementation were conducted as previously described (9). Plasmids p0380-bar and p0380-sur-gateway were used to construct gene disruption and complementation vectors. First, the 5′ and 3′ flanking fragments of genes were amplified from the WT genomic DNA with paired primers P1/P2 and P3/P4 and inserted into the BamHI-EcoRI and XbaI-SpeI sites of p0380-bar, respectively. The resultant plasmid was used to disrupt the target gene. To prepare the complementation vector, the full-length gene with promoter sequence was cloned from the WT genomic DNA with primers P7 and P8. The resulting fragment was cloned into the vector p0380-sur-gateway. The method of *Agrobacterium*-mediated transformation was used to transform the disruption vector into the WT strain and complementary vector into the gene disruption mutant. Putative deletion and complementation mutants were screened by phosphinothricin (200 μg/mL) and chlorimuron ethyl (10 μg/mL), respectively. Candidate transformants were prescreened by PCR with the primer pair P5/P6. To further confirm, Southern blotting was performed with a digoxigenin (DIG) DNA labeling and detection kit (Roche, Germany). A fragment amplified with the primers P9 and P10 was used to prepare the probes.

### Effects of gene loss on fungal phenotypes.

Conidia were harvested from fungal strains grown at 25°C on SDAY plates for 7 days and used as initial inocula in assays. All phenotypes were compared among the wild-type, gene disruption mutant, and complemented mutant strains, and the entire experiment was replicated three times (11).

### (i) Assay for ATP concentration.

Fungal strains were cultured in SDB at 25°C for 2 days and on SDAY at 25°C for 3.5 days. Mycelia were washed with phosphate-buffered saline (PBS) buffer and ground to powder in liquid nitrogen. The resulting powder was suspended in the lysis buffer from the enhanced ATP assay kit (catalog no. S0027; Beyotime Biotechnology, Shanghai, China). The ATP content was determined according to the manufacturer’s instructions. The concentration of ATP was calculated as ATP amount per gram mycelium.

### (ii) Vegetative growth on nutrients.

Conidia were suspended in 0.02% Tween 80 solution. Aliquots of 1-μL suspensions (10^6^ conidia/mL) were inoculated on the CZA plates modified with different carbon and nitrogen sources. SDAY plates were used as the control for rich medium. After the 7-day incubation at 25°C, the colony diameter on plates was measured, and its biomass was determined after drying.

### (iii) Spore formation.

For aerial development, conidial suspensions (100 μL of 10^7^ cells/mL) were inoculated on the SDAY plates and incubated at 25°C. At 7 days postincubation, mycelial discs were sampled from plates, and conidia on mycelia were suspended into 0.02% Tween 80 solution. Conidial concentrations in suspension were quantified and used to calculate conidia yield, shown as spore number per centimeter squared.

The blastospore formation was determined under submerged conditions. SDB was prepared by omitting agar in SDAY medium. Trehalose-peptone broth (TPB), mimicking insect hemolymphs, consisted of 3% trehalose and 0.5% peptone. The conidial suspension was inoculated into culture broth (conidial final concentration of 10^5^/mL) and cultured at 25°C for 2 days with shaking. The blastospore concentration was quantified and presented as spore number per milliliter culture broth.

### (iv) Response to oxidative stress.

Conidial germination and radial growth were tested on CZA plates. Conidial suspensions (1 μL, 10^6^ conidia/mL) was inoculated on the CZA media supplemented with different stress reagents, including 1 mM H_2_O_2_ and 0.02 mM menadione. The colony diameters were also measured at 7 days postincubation at 25°C. The CZA plates without stress were used as control. Blastopore formation under oxidative stress was determined by adding oxidation reagents into SDB and TPB media.

### (v) Assay for intracellular superoxide levels.

Superoxide production was estimated using the fluorescent dye dihydroethidium (DHE) obtained from Molecular Probes (Eugene, OR, USA) according to the documented method (36). Mycelia were culture in TPB and then stressed by H_2_O_2_ and menadione. The resultant mycelia were washed three times with PBS and labeled with DHE (10 μmol/L in 1% dimethyl sulfoxide [DMSO]) at 37°C for 30 min. Then, the resultant mycelia were washed three times with PBS, and the fluorescent signals were visualized under a laser scanning confocal microscope.

### Fungal virulence.

Two kinds of infection methods were used to determine fungal virulence against the larvae of Galleria mellonella. Conidia were suspended in 0.02% Tween 80 solution and adjusted to concentrations of 10^5^ and 10^7^ spore/mL, which were applied in the cuticle and injection infection methods, respectively. In cuticle infection assay, the insects were immersed in suspension for 15 s. For injection assay, a conidial suspension (5 μL) was injected into the host hemocoel. All infected insects were incubated at 25°C, and the mortality was recorded daily. The median lethal time (LT_50_) was determined by probit analysis for three replicates of each bioassay. Each treatment included 30 to 35 insects.

Additionally, fungal development in the host hemocoel was determined by quantifying the production of *in vivo* hyphal bodies in hemolymphs at 2 and 3 days postinfection.

### qRT-PCR analysis of *BbSdhC1* expression under oxidative stress.

A conidial suspension (100 μL, 10^7^ conidia/mL) was inoculated on CZA plates with H_2_O_2_ (2 mM) and menadione (0.02 mM), using CZA as control. Mycelial colonies grown on plates for 3 days at 25°C were sampled and ground into powder. Total RNAs were extracted using the RNAiso Plus reagent (TaKaRa, Dalian, China) and reverse transcribed into cDNAs using PrimeScript RT reagent kit (TaKaRa). The qRT-PCR system was established using SYBR premix Ex Taq (TaKaRa) and primer pair P13/P14 (Table S1), and analysis was performed on a Mastercycler ep realplex machine (Eppendorf, Hamburg, Germany). Under the indicated condition, the relative expression level of *BbSdhC1* was calculated using its expression level in the Δ*Bbmbp1* mutant divided by the corresponding expression level in the wild-type strain using the threshold cycle (2^−ΔΔ^*^CT^*) method (37), using the actin gene as the internal reference.

### Yeast one-hybrid analysis.

BbSdhC has been revealed as a differentially downregulated gene in the BbMbp1-mediated transcriptome. Y1H analysis was used to confirm the interaction of the BbMbp1 with the promoter region of BbSdhC as described previously (9). All manipulations were conducted according to the manual of the Matchmaker Gold yeast one-hybrid system (TaKaRa, CA, USA). In brief, the 1,250-bp promoter sequence of target gene was amplified by PCR with the primers P11 and P12. DNA fragments were cloned into the vector pAbAi to generate the bait construct. The expression vector pGADT7-Rec-BbMbp1 (9) harboring BbMbp1 was introduced into the reporter strain, which was constructed by integrating the bait vector into the Y1H Gold yeast strain. The interaction was revealed by growing the yeast transformants on selective medium in which synthetic dextrose medium lacking uridine and leucine was supplemented with 200 ng/mL aureobasidin A. All positive and negative controls were provided by the kit.

### Statistical analysis.

Difference in fluorescence intensity among strains was analyzed by Mann-Whitney test and considered significant if *P* values were <0.05. All other phenotypic measurements for the wild-type, gene disruption, and complementation strains were subjected to Student’s *t* test, and the significance was determined if *P* values were <0.05.
