# Supplementary material for: Succinate Dehydrogenase Subunit C Contributes to Mycelial Growth and Development, Stress Response, and Virulence in the Insect Parasitic Fungus Beauveria bassiana
Source: Microbiol Spectr. 2022 Aug 16;10(5):e02891-22. doi: 10.1128/spectrum.02891-22 (PMC9602434; doi:10.1128/spectrum.02891-22)
Supplement: Supplemental file 1 — Table S1; Fig. S1 to S4. Download spectrum.02891-22-s0001.pdf, PDF file, 1.7 MB [file spectrum.02891-22-s0001.pdf]

Table S1 Primers used in this study.

Primers	Paired sequences (5′–3′)*	Purpose
P1/P2	ACGAGCTGTACAAGTAACCCGGGAAGTAAAGCCGAAGAGTG/ CTCGGTACCAAGCTTGGCTGCAGGTTTGTCTTGGGTATGGG	Amplifying 5′-fragment of *BbSdhC1*
P3/P4	ACCCATGGCTCGAGTCTAGACTGTCTGGCGTGGCGTAT / GTGGCTAGCGTTAACACTAGTTCCGAGAATGAAGTCCGC	Amplifying 3′-fragment of *BbSdhC1*
P5/P6	ATCCGTCGACCTGCAGCCAAGCTTTCCGAGTAAGTAATGCTTTCTATG / ACACTAGTCAGATCTTCTAGTGTCCATAACGGAACGACTACAACTA	Cloning the *BbSdhC1* full ORF for gene complementation
P7/P8	GCATCGTGTCCACCTACC / AAGCCCGTTTGCGTCCAT	Screening transformants
P9/P10	TCGGTCGTATGATAGGATGATTT/TTAACGTGCATAGCGAGTGGAAC	Preparing *BbSdhC1* probe
P11/P12	AAGCTTGAATTCGAGCTCGGTAATCTCGCTCGGCTGTCCT / ACACTAGTCAGATCTTCTAGTGTGCTTGTAGTTCGCATTCC	Amplifying the promoter of *BbSdhC1*for Y1H test
$PL_{BbSdhC1}$ F/R	TGGGCCCGGCGCGCCGAATTCCCCCCTTACAACAACCAT/ TGGCTGCAGGTCGACGGATCCTTGATAGCCTGGTCCTGA	Amplifying *BbSdhC1* cDNA
$P_{BbSdhC2}$F/R	GACCCATGGCTCGAGTCTAGAGCACCGACCTCGCTGAAA/ GTGGCTAGCGTTAACACTAGTGTCCGTCGCCATCCTCTT	Amplifying *BbSdhC2* cDNA
P13/P14	TCAGGCGTCTCCGTCTTGAG/CAGTGATGAGCAGATGGA	qRT-PCR for *BbSdhC1*
ActinF/R	GGCAACATTGTCATGTCTGG/TTTGCTGGAAGGTGGATAGG	Reference in qRT-PCR

*: The underlined region is required for homologous recombination during plasmid construction.

Fig. S1 Sequence alignment of the SdhC homologs. Multiple sequence alignment was performed by ClustalW software. Abbreviations for fungal species are shown as follows, Sc: *Saccharomyces cerevisiae* S288C; Ca: *Candida albicans* 12C; Sp: *Schizosaccharomyces pombe*; Af: *Aspergillus fumigatus* Af293; Po: *Pyricularia oryzae* Y34; Nc: *Neurospora crassa* OR74A; Bb: *Beauveria bassiana* ARSEF 2860; Cm: *Cordyceps militaris* CM01; Bbr: *Beauveria brongniartii* RCEF 3172; Mr: *Metarhizium robertsii* ARSEF 23; Ma: *Metarhizium acridum* CQMa 102.

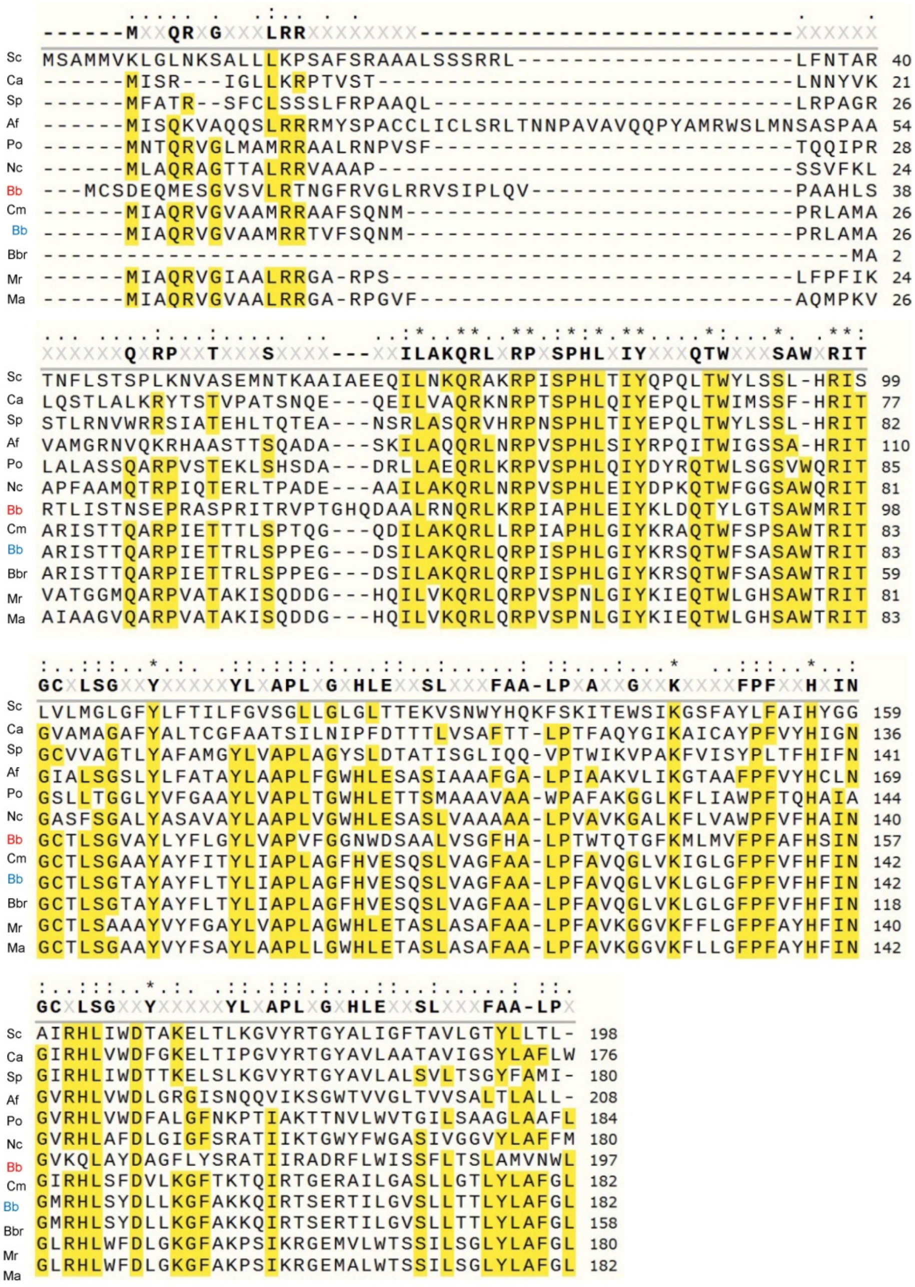

Fig.S2 Gene disruption and complementation in *B. bassiana*

(A) Schematic diagram deciphering the homologous recombination for gene disruption in fungal genome. The correct gene disruption and complemented mutants were screened by PCR reaction (B) and further confirmed by Southern blotting (C). In panel (B), lane 1, 2 and 3 are the wild-type, Δ*Bbsdhc1*, and complementation stains. Lane M: DNA marker. In panel (C), lane 1, 2 and 3 are the Δ*Bbsdhc1*, wild-type, and complementation stains. The electrophoretic positions and sizes of the DNA bands are indicated.

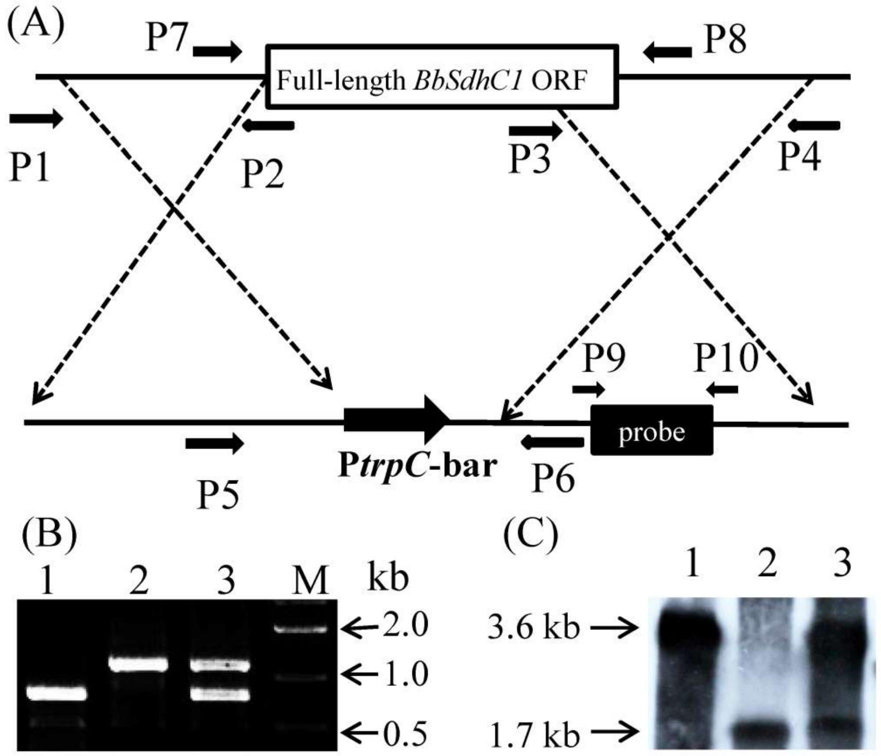

Fig. S3 ROS content in aerial mycelia of *B. bassiana*

(A) After cultured on SDAY medium for 4 days, the hyphae of Δ*Bbsdhc1* mutant, the wild-type and complemented strain were collected and stained with DHE. Green fluorescence signals of each strain were observed under laser scanning confocal microscope. (B) The fluorescence intensity among strains was subjected to Mann-Whitney analysis and significance (indicated with an asterisk) was determined when p value is less than 0.05.

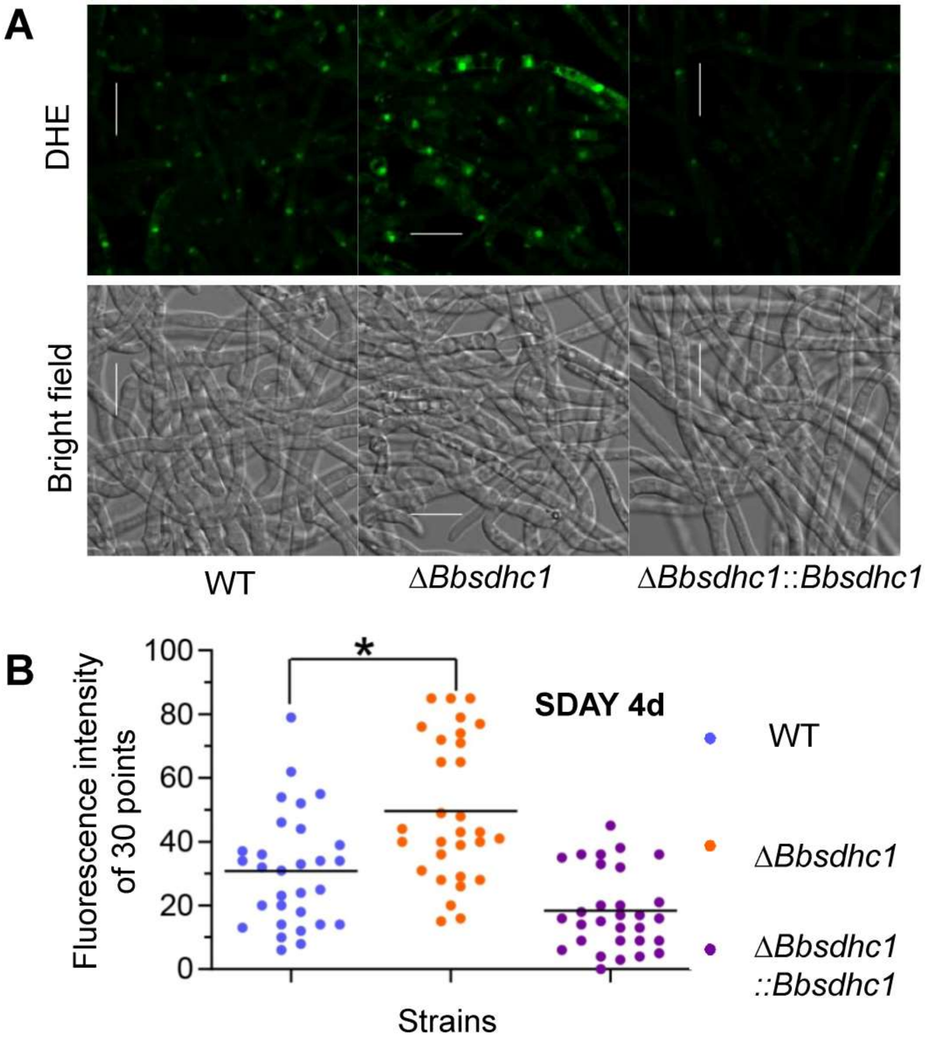

Fig. S4 Analyses of the promoter region of *BbSdhC1*. No typical *Mlu*I sites in the promoter of BbSdhC1, but the similar sites are present.

ATCTCGCTCGGCTGTCCTGGCCGTCCAAGCTGCGACTTCTTTCCAAACCCCTCCATCTGCTCTGGCGGGC
GTGACGCAGGCTGCAGTGGCGTGTGGACTGGACCAGGCGCCACAGCATTGACGCGAATGCCCTTTGG
AAGCAGCTGTTTTGCCAAGGACCTAGTGAAGGACGTGATGGCACCCTTTGTGGCCGAATAGTCTACCAT
TGCGGCAGTTCCTCTAAACGCGACCGTCGATGTGGTGTTGATTATGCTGTGATGACCATGTCAGCAGAT
GGCTTCAGTGAGGGATCCGGCATCATGGTCACGTACGATCCACCTCGTTGCATGTGAGGCAGAGCATA
TTTCGTAATGGCAAACATTTGCAAAATGTTGCTCCTGAATGTGCTCTCAACATCGTCGAGGTTTATTTCA
GCGAGGTCGGTGCACATGATTTGCTTGGATGCGTTGTTGACCAGTGTGTGAATGACACCAAACTCCTCG
ACATGCTTTTCGACAGCCTGGAGACAGGTTTTGTTGTCCATTAGATCACCCGCAACCAAGAGGCACTTTC
CGCCCTCCTTTTCCACCATTTTCTTCGTATCGTTGGCGTCTTGCTCCTCCTCTGGCAGGTACACAATCGTC
ACGTCGGAGCCTTCTCTGGCAAACAAGACGGCAATTGAGCGACCGATGCCAGAGCTGTAAAGTCAATC
AATAATTGCTAACCCAGAACGAGCAAGGGGAAGAAACTGAGCTACTCACTCGCCACCTGTAATGAGAG
CCTTGTTGCCTTTCAATTTGCCCGCAGCAAGATATTCATGGTGCTTGTCTTTGCCCTCGAGCTTAGTAGA
CTCGCTGGACGGCGCCATGTCCCTTTCGGACCTAGAAAAAAAAAACAGTTAGTGTTGCTGCTCTCATGT
ATGCATCGTGTCCACCTACC CGGGGAGATTCTGGGCTTGAGCCTGAGGAACAGGCTTGAAGCCACCAC
CTTCGGACTTGGACATGGAGAATGGTACGATAGAAAAATAGCGTGGAATAATTCTTGATGAGAATTGA
GTGTGTAACCCGGCACGGAAATTGCAAATTGATAGCCTGGTCCTGATGTGACTAGCTGGGAGTTTGAT
GAGATTGATACGTGATGGGTGCATGGCGGGCTTTTGTAGTAGACTGAGCAGACCTGGCCCATACCCAA
GACAAACAGTTTTATCCTGATTTTAACAAGTCAAATAGGCCAATGAATTCAGGGAATGCGAACTACAAG
C
